# Cost-Effectiveness Analysis of Rivaroxaban in Chinese Patients With Stable Cardiovascular Disease

**DOI:** 10.3389/fphar.2022.921387

**Published:** 2022-06-20

**Authors:** Tianyu Feng, Zhou Zheng, Shang Gao, Jiaying Xu, Pen Cao, Huanhuan Jia, Xihe Yu

**Affiliations:** School of Public Health, Jilin University, Changchun, China

**Keywords:** rivaroxaban, aspirin, cost-effectiveness, COMPASS, markov model

## Abstract

**Objective:** This study aimed to investigate the cost-effectiveness of low-dose rivaroxaban plus aspirin versus aspirin alone for patients with stable cardiovascular diseases in the China.

**Methods:** We used TreeAge 2019 to construct a Markov model to assess the direct healthcare costs and quality-adjusted life years for three therapies, namely low-does rivaroxaban plus aspirin, rivaroxaban alone, and aspirin alone. Transitional probabilities were derived from the COMPASS trial, and the costs and utilities were obtained from the Chinese Health Care Statistical Yearbook and published studies. Use the Incremental cost-effectiveness ratio to describe the results. The willingness-to-pay threshold is set at US$11,000 (China’s 2020 Gross National Product per capita).

**Result:** In patients with stable cardiovascular disease, the increased cost per quality-adjusted life year gained in the low-dose rivaroxaban combined with aspirin group compared to the aspirin alone group was US$7937.30. The increased cost per quality-adjusted life year gained in the rivaroxaban alone group versus the aspirin alone group was US$15,045.78.

**Conclusion:** A low-does rivaroxaban plus aspirin therapy may be cost-effective in the secondary prevention of stable cardiovascular disease in patients.

## Introduction

Cardiovascular diseases (CVDs) is the leading cause of death in China (Zhao et al., 2019). Inadequate secondary prevention, however, is an important reason why patients with stable CVDs repeatedly experience adverse cardiovascular events and contribute to out-of-hospital deaths (Mega et al., 2012). Aspirin is often used in clinical practice for the secondary prevention of adverse cardiovascular events in stable CVDs populations (Antithrombotic Trialists' Collaboration, 2002). Even so, the risk of adverse CVDs events in people with stable CVDs remains high (Baigent et al., 2009). This risk may be related in part to excess thrombin generation that persists beyond the acute presentation in such patients (Merlini et al., 1994). Rivaroxaban, an oral anticoagulant that directly and selectively inhibits factor Xa, has been proven to reduce CVDs mortality and the risk of stroke and myocardial infarction (MI) in patients with acute coronary syndromes (Mega et al., 2012). In the recent randomized controlled trial (RTC) entitled “Cardiovascular Outcomes in People Using Anticoagulation Strategies” (COMPASS trial), a combination of low-dose rivaroxaban (2.5 mg twice daily) and aspirin (100 mg once daily) was found to help significantly reduce the risk of major adverse cardiovascular events in patients with stable heart disease compared to aspirin alone risk of major adverse cardiovascular events in patients with stable CVDs (Eikelboom et al., 2017).

The RCT confirmed the advantage of low-does rivaroxaban plus aspirin in the secondary prevention of stable CVDs patients. However, low-dose rivaroxaban plus aspirin in the COMPASS trial was associated with more non-fatal bleeding times and the cost of rivaroxaban was much higher than that of aspirin. Rivaroxaban for stroke prevention in atrial fibrillation and secondary prevention in patients with continuing coronary syndrome has been shown to be cost-effective in studies in high-income countries and regions (Ademi et al., 2018; de Jong et al., 2019). However, the cost-effectiveness of this therapeutic schedule in low- and middle-income countries, particularly in mainland China, is uncertain.

## Materials and Methods

### Model Structure

**FIGURE 1:**
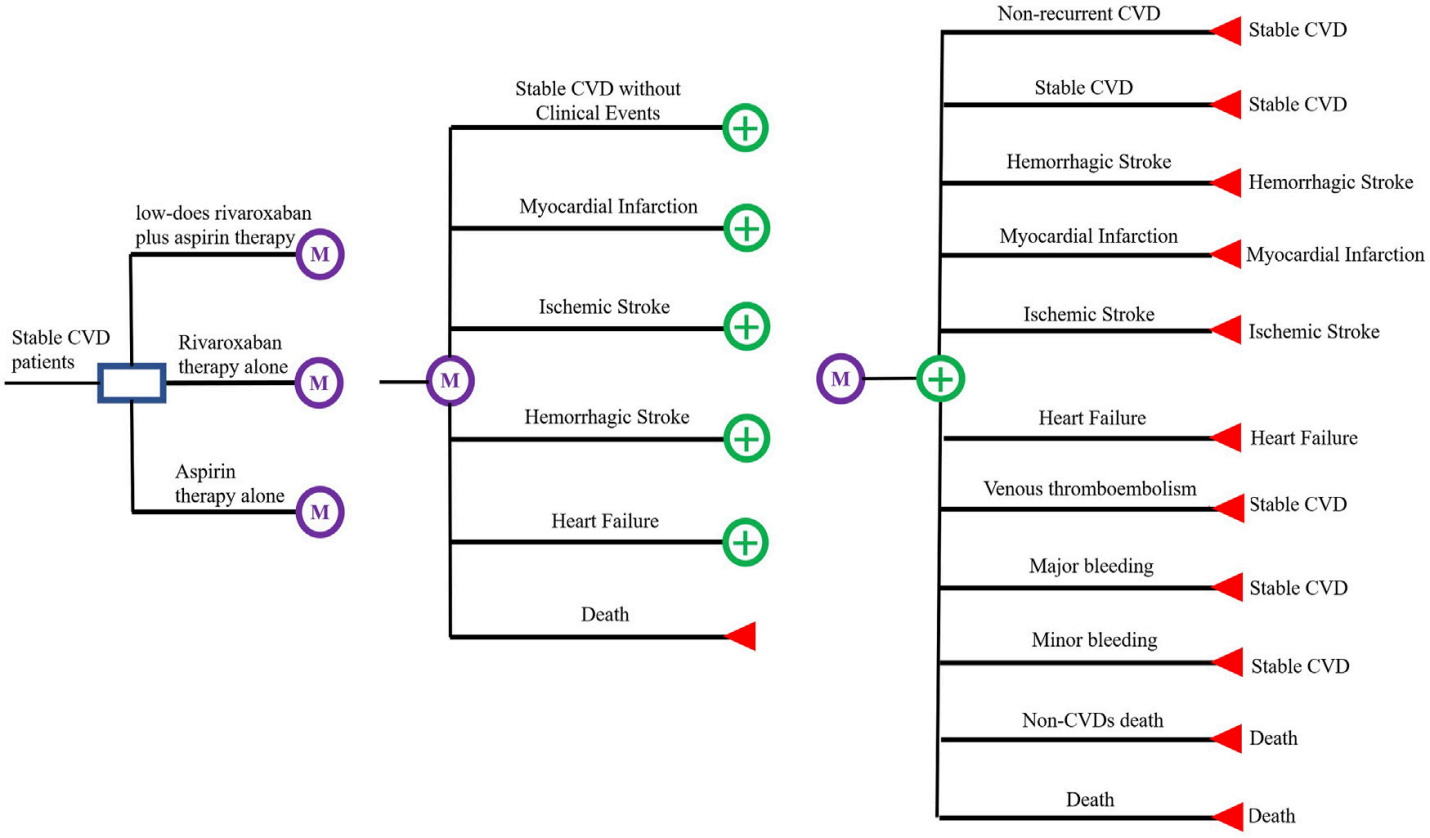
Schematic representation of the Markov model.

The main outcome of interest in this study is the incremental cost-effectiveness ratio (ICER) in terms of the cost per quality-adjusted life year (QALY) gained and the cost per year of life saved (YoLS) (Ademi et al., 2018). A Markov decision model was developed to assess the cost-effectiveness of three treatment regimens, low-dose rivaroxaban (2.5 mg twice daily) plus aspirin (100 mg once daily); rivaroxaban alone (5 mg twice daily); and aspirin alone (100 mg once daily), for secondary prevention in patients with stable CVDs. A Markov model with a cycle length of 1 month was constructed from the results of the COMPASS trial (Eikelboom et al., 2017). For each cycle in the Markov model there are two components, acute event state and pre-acute event state (Harrington et al., 2013; Krejczy et al., 2014). Pre-acute events state include six states, including ‘stable CVDs without clinical events’, ‘myocardial infarction (MI)’, ‘ischemic stroke (IS)’, ‘hemorrhagic stroke (HS)’, ‘heart failure (HF)’ and ‘death’. Acute events state includes ‘non-recurrent CVDs’, ‘recurrent CVDs (MI, IS, HS and cardiovascular death)’, ‘HF’, ‘venous thromboembolism (VTE)’, ‘major bleeding’, ‘minor bleeding’ and ‘death.’ Also included in each state is the patient’s non-CVDs mortality event. All analyses were operated by TreeAge 2019 and Microsoft Excel. The patient can transfer between the states by pressing the arrow, as shown in Figure 1.

### Model Population

Assume a cohort population size of 1,000 people and set their initial age at 65 years, with a maximum cohort age of 78 years (the life expectancy of the Chinese population in 2020). They have coronary artery disease (CAD), peripheral artery disease (PAD) or a combination of both. They are not at high risk of bleeding, recent stroke or previous hemorrhagic or lacunar stroke, severe heart failure or advanced chronic kidney disease (estimated GFR <15 ml/min). In addition, it was assumed that they were not receiving antiplatelet therapy, anticoagulation or other antithrombotic treatment and did not have non-CVDs associated with poor prognosis.

### Transition Probabilities

The transition probabilities used in this study were derived from the COMPASS trial (Eikelboom et al., 2017), which followed participants for an average of 23 months, and were converted into monthly transition probabilities using the formula below (Sonnenberg and Beck, 1993). All-cause mortality probabilities were obtained from the Chinese Yearbook of Health Statistics (Guo, 2020a). All transition probabilities were assumed to remain constant over the observation time and each subject in the model was followed up to their death based on the transition probabilities. Age-dependent non-CV deaths were all from the Report on China’s Cause of Death 2020, which is published by the China Center for disease Control and Prevention.

$$r=-\frac{1}{t}\ln\left(S\right)$$

$$P=1-e^{-r*T}$$

Among them, S is the rate, t is the time, and P is the transition probability converted into every 1 month (Table 1).

**TABLE 1:** Monthly transitional probability of the clinical events.

	Riv + Aspirin	Asp	Riv	Distribution	Reference
Myocardial infarction	0.000853589	0.000988299	0.000876724	Beta	COMPASS (Mega et al., 2012)
Ischemic stroke	0.0003242	0.000633898	0.000436152	Beta	COMPASS (Mega et al., 2012)
Hemorrhagic stroke	0.000713162	0.000477143	0.000128952	Beta	COMPASS (Mega et al., 2012)
Venous thromboembolism	0.000118923	0.000195948	0.000172021	Beta	COMPASS (Mega et al., 2012)
Heart failure	0.000945657	0.000924984	0.000920541	Beta	COMPASS (Mega et al., 2012)
Major bleeding	0.001213227	0.000629063	0.001164774	Beta	COMPASS (Mega et al., 2012)
Minor bleeding	0.004166582	0.002464437	0.003685666	Beta	COMPASS (Mega et al., 2012)
Cardiovascular death	0.000766538	0.000978553	0.000940029	Beta	COMPASS (Mega et al., 2012)
All-cause death	0.001511842	0.001839395	0.001924851	Beta	COMPASS (Mega et al., 2012)
Riv, rivaroxaban alone; Asp, aspirin alone; Riv + Asp: low-does rivaroxaban plus aspirin					

### Disease Costs and Health Utility

This analysis was conducted from the perspective of the Chinese healthcare system. In the analysis medical costs are taken from the Chinese Health Care Statistical Yearbook (Guo, 2020a) and the costs of rivaroxaban and aspirin are the market retail prices of the drugs (Table 2). Data on health utility values are taken from relevant data in the University of Oxford’s Health Economics Strategy (Table 3) (Briggs et al., 2006). The length of each cycle of the model was 1 month (30 days), so the model was run for a total of 156 cycles. The total cost is accumulated by multiplying the cohort size with the sum of the costs for each health state. Total costs are accumulated by multiplying the cohort size by the sum of the costs for each health state. The QALYs for each cycle are calculated using the utility value associated with each health state multiplied by the proportion of years lived in that state. The total QALY and life year is the accumulation of QALY and life year values over all cycles. icer includes cost per QALY gained and cost per life year saved, calculated by dividing the incremental cost by the incremental QALY and life year.

**TABLE 2:** Input parameters for the costs in the model.

Variables	Cost	Range	Distribution	Reference
Monthly costs of rivaroxaban (5 mg twice per day)	68.5	54.8∼82.2	Gamma	Local data
Monthly costs of aspirin (100 mg once per day)	2.5	2∼3	Gamma	Local data
Monthly costs of rivaroxaban (2.5 mg twice per day)	34	27.2∼40.8	Gamma	Local data
Myocardial infarction	4,127	3,301.6∼4,952.4	Gamma	Guo, (2020a)
Ischemic stroke	1,344	1,075.2∼1,612.8	Gamma	Guo, (2020a)
Hemorrhagic stroke	2,735	2,188∼3,282	Gamma	Guo, (2020a)
Venous thromboembolism	1,252.5	1,002∼1,503	Gamma	Guo, (2020a)
Heart failure	1,183	946.4∼1,419.6	Gamma	Guo, (2020a)
Major bleeding	716	572.8∼859.2	Gamma	Guo, (2020a)
Minor bleeding	286	228.8∼343.2	Gamma	Guo, (2020a)

**TABLE 3:** Input parameters for the utilities.

Variables	Utility Estimates	Standard Error	Distribution	Reference
Stable cardiovascular disease	0.738	0.0153	Beta	Briggs et al. (2006)
Decrement for age	−0.0016	0.0001	Beta	Briggs et al. (2006)
Myocardial infarction	0.704	0.0658	Beta	Briggs et al. (2006)
Ischemic stroke	0.65	0.0954	Beta	Briggs et al. (2006)
Hemorrhagic stroke	0.65	0.0954	Beta	Briggs et al. (2006)
Venous thromboembolism	0.727	0.0663	Beta	Briggs et al. (2006)
Heart failure	0.636	0.1015	Beta	Briggs et al. (2006)
Decrement for major bleeding	−0.1814	0.013	Beta	Briggs et al. (2006)
Decrement for minor bleeding	−0.0582	0.017	Beta	Briggs et al. (2006)

### Model Outcome

Notably, the following is according to the recommendation of the World Health Organization (WHO) for the evaluation of pharmacoeconomic (Eichler et al., 2004): ICER <1 fold of gross domestic product (GDP) per capita, the increased cost is completely worth it and very cost-effective; 1 fold of GDP per capita < ICER <3 fold of GDP per capita, the increased cost is acceptable and cost-effective; ICER >3 fold of GDP per capita, the increased cost is not worth it and not cost-effective. China’s GDP per capita in 2020 (Guo, 2020b) (US$11,000) is used as the threshold for willingness-to-pay (WTP) (Marseille et al., 2014; Bertram et al., 2016).

### Treatment Cost

The cost of treatment with rivaroxaban is based on the latest national negotiation price in 2020 (Table 2). As rivaroxaban is currently only available in 10, 15 and 20 mg from the Chinese Essential Drug List, the cost per mg of 10 mg rivaroxaban was adjusted in this study: 2.5 mg rivaroxaban twice daily cost US$1.14 per day; 5 mg rivaroxaban twice daily cost US$2.28 per day. The cost of aspirin (100 mg per day) also comes from the National Essential Medicines List: US$0.08. Costs of treatment are discounted at an annual rate of 5% according to the Chinese Pharmacoeconomic Assessment Guidelines (Wang et al., 2015).

### Uncertainty and Scenario Analysis

Many of the parameters used in this study have considerable uncertainty. For this reason, one-way sensitivity analysis and probabilistic sensitivity analysis were conducted to assess the effect of uncertainty on the robustness of the results (Moayyedi, 2007).

The impact of each input parameter on the results was assessed by a deterministic sensitivity analysis (DSA) with a one-way sensitivity analysis (± 20% of the input parameter), using tornado plots to present the results for each parameter.

Probabilistic sensitivity analysis (PSA) was used to determine the uncertainty of the input parameters (Briggs et al., 2003). PSA was performed using Monte Carlo simulation simulations with 1,000 iterations. Each parameter was specified a certain distribution, where the mean of the distribution is typically equal to the point estimate. where the transfer probability uses and the utility value uses the Beta distribution and the treatment cost using the gamma distribution. Additional scenario analyses were undertaken to explore other model assumptions:

1) The time range is set at 2, 5 and 13 years.
2) Different discount rates are set: 3 and 5%.
3) Halve the cost of rivaroxaban.

The Cost-Effectiveness Acceptable Curve (CEAC) and the Incremental Cost and Incremental Quality Adjusted Life Year cost-effectiveness (CE) plane are used to present the results of the probabilistic sensitivity analysis.

## Results

### Cost-Effectiveness Analysis

Table 4 reports the base case analysis. Subjects in the low-does rivaroxaban plus aspirin cohort spent an average of 11.72 years at an average total cost of US$4,909.59; subjects in the aspirin group lived an average of 11.45 years at an average total cost of $1,454.32; subjects in the rivaroxaban group lived an average of 11.60 years at an average total cost of $7771.67. The ICERs for low-dose rivaroxaban and rivaroxaban groups compared to aspirin are US$7937.30 and US$15045.77 for aspirin respectively. The low-dose rivaroxaban and aspirin combination regimens are below China’s 2020 GDP per capita of US$11,300. the low-dose rivaroxaban regimen is more cost effective in comparison. Compared to the aspirin group, the ICERs per YoLS gained were US$12,494.79 and US$42,192.37 for the low-dose rivaroxaban and rivaroxaban groups, respectively.

**TABLE 4:** The result of base-case cost-effectiveness analysis and scenario sensitivity analyses.

		Cost	ICER cost	EFF	ICER EFF	ICER
	Asp	1,445.06		48.20		
	Riv	7773.92	6,328.86	48.63	0.42	15045.78
Base-case analysis	Riv + Asp	4,818.65	3,373.59	48.63	0.43	7937.30
	Asp	253.39		16.61		
	Riv	1,352.58	1,099.19	16.65	0.04	27722.28
Time horizon (2 years)	Riv + Asp	833.96	580.57	16.66	0.04	13609.23
	Asp	617.61		34.07		
	Riv	3,284.32	2,666.71	34.25	0.18	14622.52
Time horizon (5 years)	Riv + Asp	2044.19	1,426.59	34.25	0.18	7822.49
	Asp	1,488.72		47.92		
	Riv	7994.24	6,505.51	48.40	0.48	13553.72
Discounting rate (3%)	Riv + Asp	4,943.66	3,454.94	48.42	0.50	6,953.40
	Asp	1,451.16		47.84		
	Riv	4,374.09	2,922.93	48.26	0.42	6,902.18
Half monthly costs of rivaroxaban	Riv + Asp	2,872.88	1,421.73	48.24	0.41	3,503.17
QALY, quality-adjusted life-year; ICER: incremental cost-effectiveness ration; Riv, rivaroxaban alone; Asp, aspirin alone; Riv + Asp: low-does rivaroxaban plus aspirin						

Patients using low-dose rivaroxaban in combination with aspirin achieved more total QALYs and a lower number of adverse events compared to those receiving aspirin alone or rivaroxaban alone.

### Sensitivity Analyses

**FIGURE 2:**
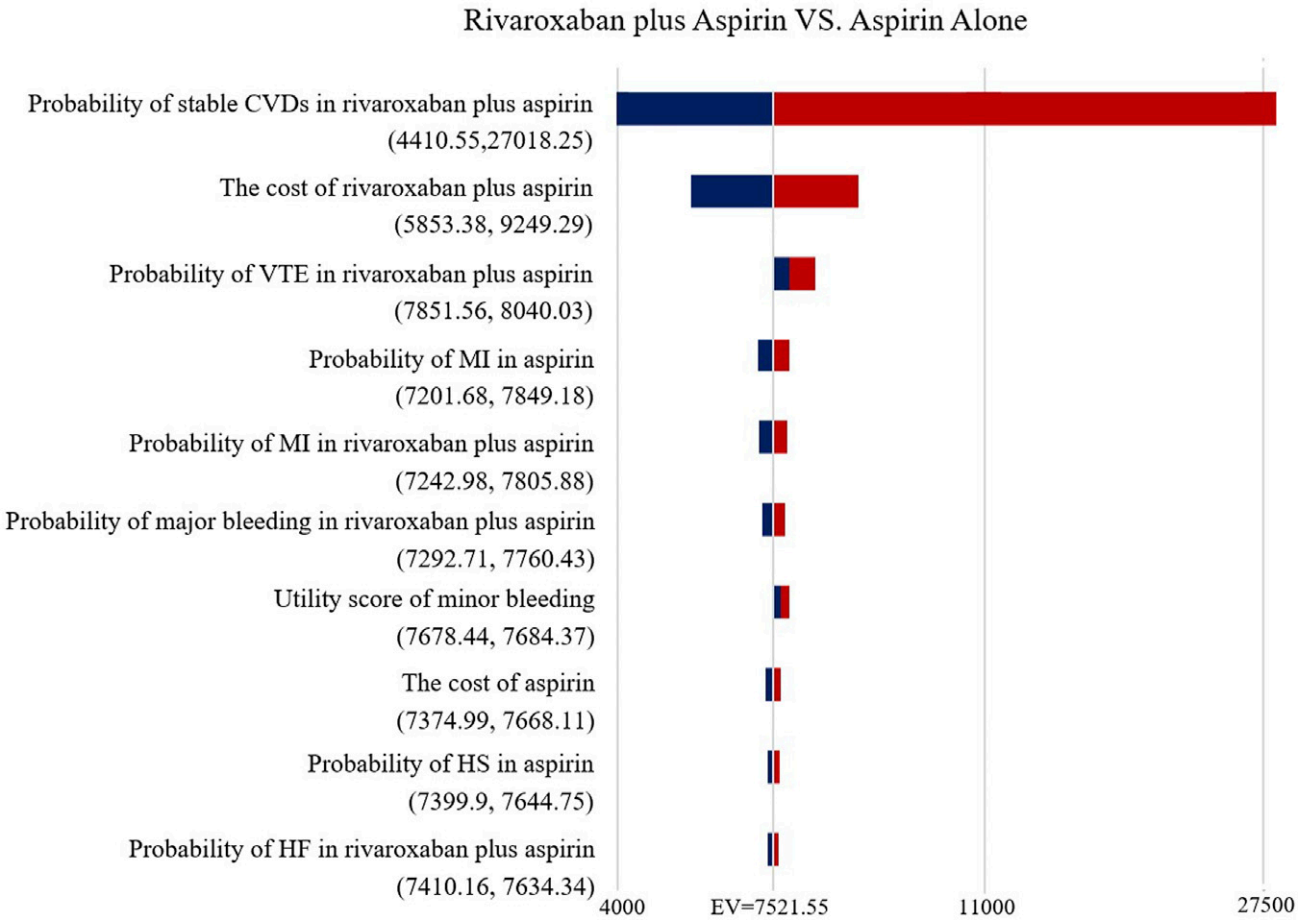
Tornado diagram showing the deterministic sensitivity analysis of the Markov model simulation (Low-does rivaroxaban plus aspirin group vs. Aspirin alone group).

**FIGURE 3:**
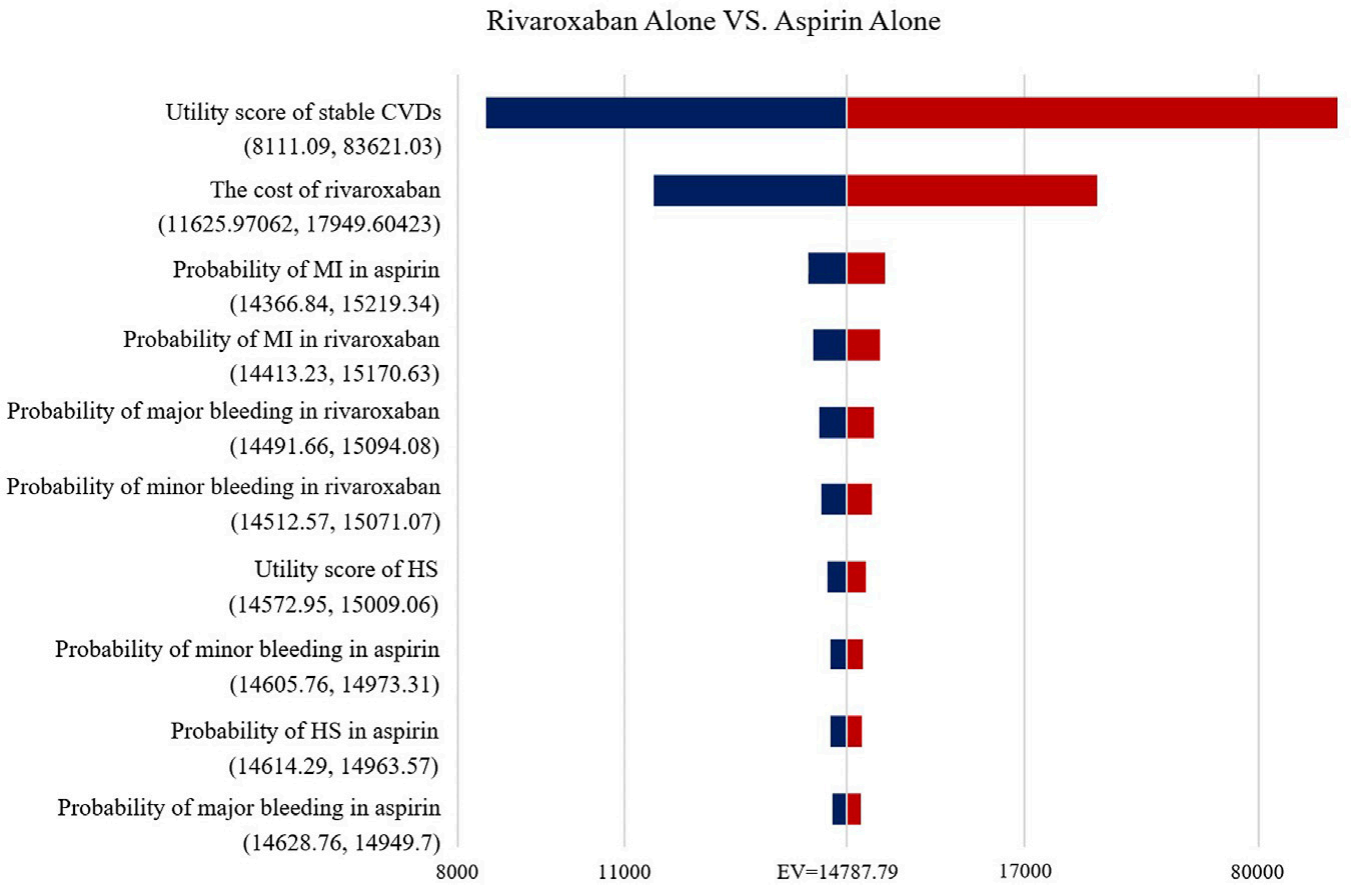
Tornado diagram showing the deterministic sensitivity analysis of the Markov model simulation (Rivaroxaban alone group vs. Aspirin alone group).

The Tornado diagram (Figure 2) demonstrates the one-way sensitivity analysis for the low-dose rivaroxaban and aspirin groups. The ICER was within three times the GDP per capita when all parameters were varied within the range of variation. Utility score of stable CVDs in the one-way sensitivity analysis for the rivaroxaban and aspirin groups had a greater impact on the results, well over three times GDP per capita, but the impact of other parameters was not significant (Figure 3).

**FIGURE 4:**
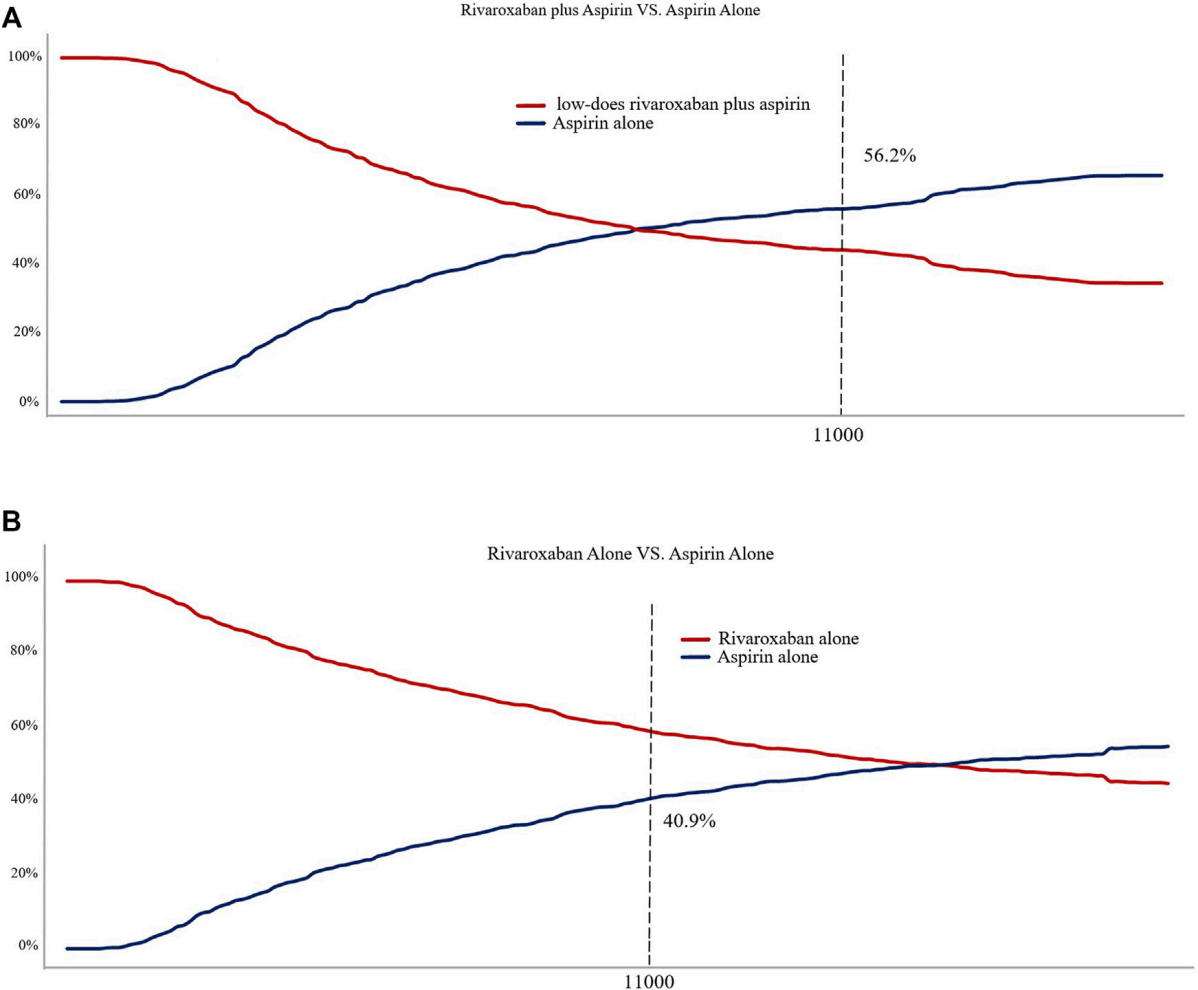
**(A)** Cost-effectiveness acceptability curve showing the maximum willingness to pay and the corresponding probability of cost-effectiveness for Low-does rivaroxaban plus aspirin group vs. Aspirin alone group. **(B)** Cost-effectiveness acceptability curve showing the maximum willingness to pay and the corresponding probability of cost-effectiveness for Rivaroxaban alone group vs. Aspirin alone group.

**FIGURE 5:**
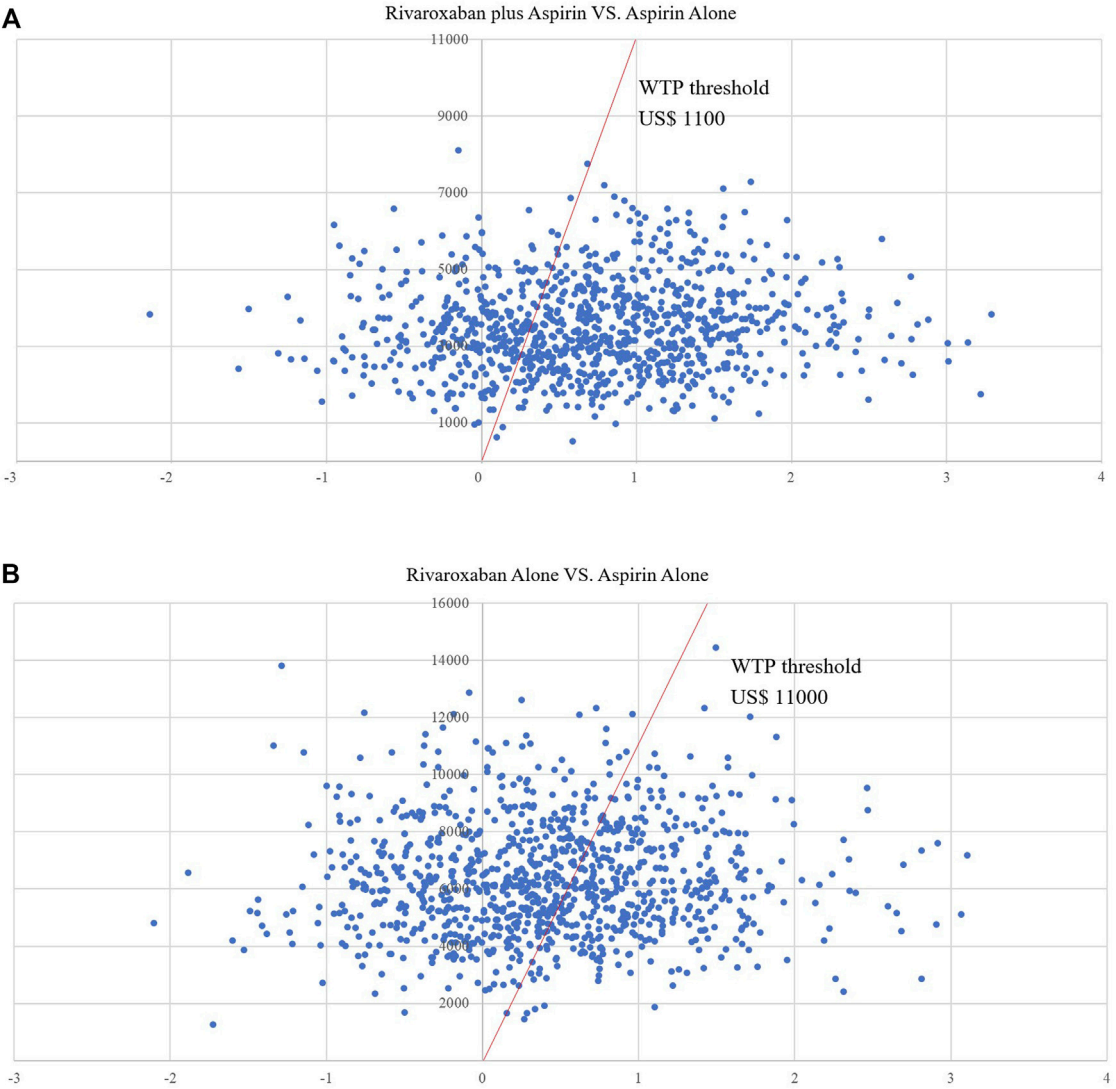
**(A)** Scatter plot showing the incremental costs and incremental quality-adjusted life-year of a thousand simulations for Low-does rivaroxaban plus aspirin group vs. Aspirin alone group. **(B)** Scatter plot showing the incremental costs and incremental quality-adjusted life-year of a thousand simulations for Rivaroxaban alone group vs. Aspirin alone group.

Figure 4 shows the CEAC for the two prevention options compared to aspirin. the CEAC at a WTP of $110,000 shows probabilities of 56.2 and 40.9% for the low dose rivaroxaban and rivaroxaban groups respectively. Figure 5 is the CE plane of the PSA results based on 1,000 Monte Carlo simulations, where the scatter is mainly in the first quadrant and mostly below the WTP threshold line. the PSA results are similar to the basic analysis: the low-dose rivaroxaban regimen is more cost effective.

### Scenario Analyses

Based on the scenario analysis, in the low-dose rivaroxaban and rivaroxaban alone groups compared to the aspirin group, the ICER decreased gradually with increasing duration of dosing and the decrease in ICER was very significant when the price of rivaroxaban was halved. The results of the scenario analysis are presented in Table 4.

## Discussion

To our knowledge this is the first cost-effectiveness analysis to assess low-does rivaroxaban plus aspirin therapy compared to aspirin alone in the treatment of patients with stable CVDs in mainland China. Our study showed that low-dose rivaroxaban in combination with aspirin was more cost-effective than treatment with aspirin alone, with an ICER of $7937.3 per QALY gained.

In the base case analysis rivaroxaban plus aspirin therapy delivered an average of 0.43 additional QALYs over 13 years for 1,000 patients over aspirin therapy, with an ICER per QALY gained below China’s GDP per capita in 2020, and the best benefit from rivaroxaban plus aspirin therapy in a range of scenario analyses. This suggests that the cost effect of rivaroxaban plus aspirin therapy is superior to that of aspirin therapy in patients with stable CVDs in the base case.

In China 2.5 mg rivaroxaban is not yet available and the ICER per QALY gained will be further reduced if the cost of the twice-daily regimen is lower in the future when 2.5 mg rivaroxaban is available than the cost per mg assumption set in this study.

To our knowledge this is the first cost-effectiveness analysis to evaluate low-does rivaroxaban plus aspirin therapy in the treatment of patients with stable CVDs in China. The COMPASS trial enrolled 27,395 patients with stable CVD from 602 centers in 33 countries and currently provides the highest level of evidence on the efficacy of rivaroxaban plus aspirin for secondary prevention of CVD. The data for this study were obtained from the COMPASS trial, which enrolled 27,395 patients with stable CVD from 602 centers in 33 countries. the COMPASS trial currently provides the highest level of evidence on the efficacy of rivaroxaban plus aspirin for secondary prevention of CVD. The use of data from the COMPASS trial as parameters for a Markov model makes this study highly representative of the target population (de Jong et al., 2019; Zomer et al., 2019; Soini et al., 2020). Low-does rivaroxaban plus aspirin therapy is cost-effective in preventing adverse cardiovascular events compared with aspirin alone in high-income countries. However, the ICER values per QALY obtained in the rivaroxaban plus aspirin group in these studies were higher than the results of this study. The main reason for this is the relatively low cost of treatment for stable CVDs and rivaroxaban in mainland China, resulting in a lower ICER for rivaroxaban plus aspirin therapy in China compared to higher income countries. For example, the cost of treatment for rivaroxaban in Australia is US$1,205 per year (Zomer et al., 2019). The cost-effectiveness of low-dose rivaroxaban in combination with aspirin in other low- and middle-income countries and regions cannot be determined due to the lack of cost-effectiveness analyses in other low- and middle-income countries and regions.

As the economics of low-does rivaroxaban plus aspirin therapy regimens have not been evaluated in China, we compared cost-effectiveness studies of relevant regimens with rivaroxaban in comparisons of other anticoagulants (Dong et al., 2020; Wei et al., 2021). A cost-effectiveness analysis based on mainland China showed that rivaroxaban proved to be more cost-effective in comparison with dabigatran etexilate, enoxaparin and warfarin under similar assumptions (Zomer et al., 2019). In our study we found that rivaroxaban alone was not cost effective compared to aspirin, therefore we believe that low-does rivaroxaban plus aspirin therapy is cost-effective in preventing all types of adverse cardiovascular events compared to other anticoagulants.

In the scenario analysis, we observed the cost-effectiveness of the three treatments at different dosing lengths by varying the cycle. In this study, we observed the cost-effectiveness of the three treatments at different dosing lengths by varying the cycle. The ICER per QALY gained for the rivaroxaban plus aspirin group decreases over time, as has been widely demonstrated in studies in other countries and regions (Zomer et al., 2019; Soini et al., 2020; Lee et al., 2021). This suggests that the use of rivaroxaban plus aspirin therapy as a long-term secondary prevention drug for stable CVDs would be more cost-effective. However, it is worth noting the increased probability of non-fatal major bleeds in the rivaroxaban plus aspirin group in the COMPASS trial and in the Australian study. Reducing non-fatal major bleeds may further reduce the ICER per QALY gained in the rivaroxaban plus aspirin therapy.

There are also some unavoidable limitations to this study. The length of observation in the COMPASS trial was only 2 years, and the observed benefits of rivaroxaban plus aspirin during this time are hardly sustainable over a long period of time (Liew et al., 2002; Briggs et al., 2006). While rivaroxaban plus aspirin therapy is still cost-effective over a 2-year period in the scenario analysis, any negative factors in the real world will affect cost-effectiveness. Secondly, the impact of non-adherence on the cost-effectiveness of drugs in the real world is real, although it is generally accepted that this impact is very limited for cost-effectiveness analysis (Ademi et al., 2018). To compensate for this, this study uses uncertainty analysis to determine the effect of changes in parameters on the final results, which goes to some extent to compensate for the effects of these experimental limitations. Thirdly, the results of the COMPASS trial were limited to non-fatal major bleeds, and differences in the site of bleeding can lead to differences in the final cost of treatment and therefore have some impact on cost-effectiveness (He et al., 2016). In addition, we acknowledge that clinical trial populations are often not representative of ‘real-world’ populations, notably with their being selected for having high risk of the conditions of interest.

The inclusion of rivaroxaban in the secondary prevention programme for stable CVD in China is still in the exploratory stage. Further economic evaluation in real-world settings is necessary for future policy makers to improve secondary prevention programmes for stable CVD.

## Conclusion

In summary, our analysis provides the cost-effectiveness of adding low-dose rivaroxaban to a prophylactic regimen for patients with chronic CVDs compared to a conventional aspirin prophylaxis regimen. The use of low-does rivaroxaban plus aspirin for secondary prevention in patients with stable CVDs should be considered cost-effective from the perspective of the Chinese public healthcare system. This study provides evidence for the cost-effectiveness of low-does rivaroxaban plus aspirin therapy for secondary prevention in patients with stable CVDs in low- and middle-income countries and regions.

## Data Availability

The original contributions presented in the study are included in the article/Supplementary Material, further inquiries can be directed to the corresponding author.
